# β-cyclocitral as a cross-species mediator of abiotic stress signaling: insights and future directions toward crop improvement

**DOI:** 10.3389/fpls.2025.1646314

**Published:** 2026-01-23

**Authors:** Grace Lachica, Prakash Basnet, Antonio Laurena, Eureka Teresa Ocampo, Ik-Young Choi

**Affiliations:** 1 Department of Agriculture and Life Industry, Graduate School, Kangwon National University, Chuncheon, Republic of Korea; 2 Philippine Genome Center - Program for Agriculture, Office of the Vice Chancellor for Research and Extension, University of the Philippines Los Baños, Laguna, Philippines; 3 Graduate School, University of the Philippines Los Baños, Laguna, Philippines; 4 Institute of Crop Science, College of Agriculture and Food Science, University of the Philippines Los Baños, Laguna, Philippines; 5 Department of Smart Farm and Agricultural Industry, Kangwon National University, Chuncheon, Republic of Korea

**Keywords:** abiotic stress, apocarotenoid, β-cyclocitral, soybean, drought stress, molecular signaling

## Abstract

Abiotic stresses such as drought, salinity, heavy-metal toxicity, and photooxidative damage severely constrain global crop productivity, a challenge intensified by ongoing climate change. The apocarotenoid β-cyclocitral (β-CC), produced via both carotenoid cleavage dioxygenase (CCD)-mediated and reactive oxygen species (ROS)-driven oxidation, has emerged as a conserved signaling molecule that enhances plant adaptation to environmental stress. β-CC mitigates oxidative damage, promotes root system remodeling, and activates detoxification pathways through ABA-independent mechanisms involving the transcriptional regulators MBS1 and SCL14. Its oxidized derivative, β-cyclocitric acid (β-CCA), extends this signaling framework by modulating the cyclin kinase inhibitor SMR5 and the cytochrome P450 gene CYP81D11, thereby strengthening photosynthetic capacity, ROS control, and developmental reprogramming under drought and high-light stress. Beyond vegetative responses, β-CC also enhances seed vigor and longevity through apocarotenoid-dependent regulation of antioxidant activity and aquaporin expression. Comparative studies across *Arabidopsis*, rice, tomato, quinoa, and peach reveal both conserved and species-specific outcomes, underscoring the versatility of β-CC/β-CCA signaling. The broad occurrence of these apocarotenoids highlights their potential as natural biostimulants and molecular tools for improving stress resilience in crops. Although direct studies in soybean remain limited, conserved orthologs and signaling components point to promising translational opportunities. Future research should clarify the dynamics of β-CC and β-CCA accumulation, validate conserved gene networks such as MBS1/SCL14/CYP81D11, and develop stable, field-compatible delivery systems. Integrating mechanistic and physiological insights from model species will accelerate the application of β-CC-based strategies for climate-resilient agriculture.

## 1 Introduction

Abiotic stresses, such as drought, salinity, and extreme temperatures, pose significant threats to global agriculture by severely hampering plant growth and reducing crop yields. Climate change has intensified these constraints through more frequent and severe weather events, leading to extensive soil degradation, salinization, and desertification (Prăvălie et al., 2021). Globally, abiotic stress can reduce crop yields by more than 60% and recent estimates attribute 58% of agricultural losses between 2017 and 2022 to natural disasters such as droughts and floods (Boyer, 1982; Daryanto et al., 2016; Webber et al., 2018; FAO, 2023). As the global population is projected to reach 10.3 billion by 2080, improving crop resilience to these environmental pressures is an urgent priority for food security (United Nations, 2025).

Plants activate complex physiological and biochemical adaptations to abiotic stress, including osmotic adjustment, antioxidant activation, and hormonal regulation (Sánchez-Bermúdez et al., 2022; Zhang et al., 2023). Among these adaptive mechanisms, carotenoid-derived apocarotenoids have attracted particular attention for their dual roles as antioxidants and signaling molecules. Under oxidative stress conditions, the degradation of carotenoids produces diverse apocarotenoids that participate in developmental regulation and stress adaptation (Ramel et al., 2012b; Zheng et al., 2021b; Mitra and Gershenzon, 2022; Stra et al., 2023). Classic examples include abscisic acid (ABA) and strigolactones (SLs), which regulate drought tolerance, shoot branching, and root development (Sharp and LeNoble, 2002; Gomez-Roldan et al., 2008; Dodd, 2013; Harris, 2015; Lanfranco et al., 2018; Barbier et al., 2019).

In recent years, β-cyclocitral (β-CC), a volatile aromatic apocarotenoid (2,6,6-trimethyl-1-cyclohexene-1-carboxaldehyde) derived from β-carotene oxidation, has emerged as a conserved signaling molecule across diverse photosynthetic and non-photosynthetic lineages. Beyond its observed roles in vascular plants, β-CC has been detected in cyanobacteria, microalgae, lichens, mosses, and fungi, suggesting that β-carotene cleavage and its signaling derivatives arose early in evolution (Saritas et al., 2001; García-Plazaola et al., 2017; Arii et al., 2021). In fungi, β-CC has been associated with distinctive flavor profiles, highlighting its sensory significance. In single-celled photosynthetic organisms, including cyanobacteria and microalgae, volatile organic compounds (VOCs) such as β-CC mediate cell-to-cell communication (Roach et al., 2022; Zuo, 2023). In bryophytes, β-CC has been implicated in allelopathic interactions, where moss or lichen extracts influence neighboring bryophytes or vascular plants (Vicherová et al., 2020; Matić et al., 2024). Within higher plants, β-CC participates in photooxidative stress mitigation, root architecture modulation, and herbivore deterrence, while also triggering programmed cell death and detoxification pathways under high-light or oxidative stress conditions (Zorn et al., 2003; García-Plazaola et al., 2017; Roach et al., 2022; Zuo, 2023; Wang et al., 2024).

Complementing these functions, the oxidized and water-soluble derivative β-cyclocitric acid (β-CCA) has recently been identified as a downstream product of β-CC oxidation in planta (D’Alessandro et al., 2019; Braat et al., 2023). β-CCA accumulates under drought and light stress and retains signaling activity, activating detoxification and cell-cycle regulators such as SCL14 and SMR5. Together, β-CC and β-CCA form a metabolic and signaling continuum that connects carotenoid oxidation to redox balance, growth modulation, and environmental adaptation across taxa. This evolutionary conservation underscores that β-CC is not merely a by-product of carotenoid degradation but a pivotal mediator of cellular communication and stress resilience throughout the plant kingdom and beyond.

Together, these observations highlight β-CC and its oxidized derivative β-CCA as evolutionarily conserved mediators of stress signaling, spanning photosynthetic and non-photosynthetic organisms alike. Despite increasing evidence of their diverse physiological roles, the biochemical origins and signaling dynamics of these molecules remain incompletely resolved. A clearer understanding of how β-CC is generated, interconverted with β-CCA, and incorporated into plant stress networks is therefore essential for interpreting their functions across species. The following sections synthesize current knowledge on the biosynthetic pathways and regulatory mechanisms underlying β-CC production and signaling, providing a framework for its potential integration into crop stress-adaptation research.

## 2 Biosynthesis of β-CC in vascular plants

**Figure 1:**
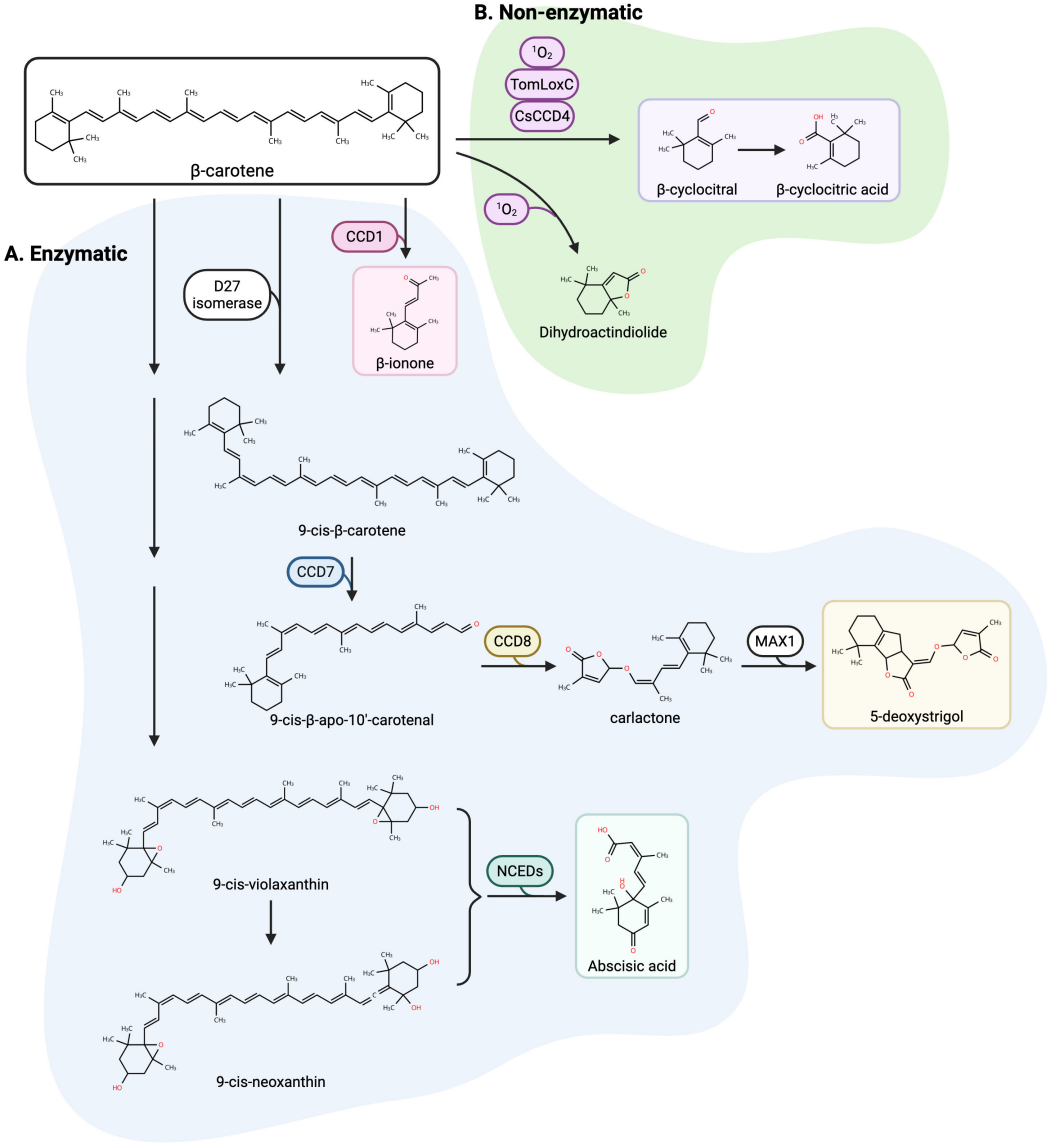
Enzymatic and nonenzymatic oxidation of β-carotene. The oxidative cleavage of β-carotene produces a variety of apocarotenoids implicated in plant responses to abiotic stress. **(A)** Enzymatic cleavage pathways: strigolactone (SL) biosynthesis: β-carotene is isomerized by D27 to 9-cis-β-carotene and then cleaved sequentially by CCD7 and CCD8 to produce carlactone, a precursor for SLs (e.g., 5-deoxystrigol) via MAX1; abscisic acid (ABA) biosynthesis: 9-cis-violaxanthin and 9-cis-neoxanthin, oxidative products of β-carotene, can be cleaved by NCEDs to generate xanthoxin, the precursor for ABA; volatile apocarotenoid production: CCD1 and CCD4 can cleave β-carotene to yield β-ionone and dihydroactinidiolide, whereas, in tomatoes and saffron, lipoxygenase (LOXC) and CCD4 contribute to β-cyclocitral (β-CC) formation. **(B)** Nonenzymatic oxidation: reactive oxygen species (ROS) mediate β-carotene breakdown into volatile apocarotenoids, including β-CC and β-cyclocitric acid (β-CCA). Created in BioRender. Lachica, G. (2025) https://BioRender.com/26d0q3r.

Carotenoids comprise a diverse group of over 750 lipophilic isoprenoid pigments characterized by a C_40_ backbone with conjugated double bonds (Cuttriss et al., 2011). This unique structure confers both photoprotective capabilities and oxidative susceptibility. In photosynthetic organisms, carotenoids serve essential roles in light absorption within the blue-green spectrum and excited chlorophyll state quenching via electron transfer, while also functioning as antioxidants and contributing to pigmentation (Mozzo et al., 2008; Hashimoto et al., 2016; Sun et al., 2022). Their structural reactivity enables oxidative cleavage into bioactive apocarotenoids through two primary pathways: enzymatic and non-enzymatic (**Figure 1**).

The enzymatic pathway centers on carotenoid cleavage dioxygenases (CCDs), which mediate specific oxidative cleavage by inserting oxygen atoms between carbon-carbon double bonds (Giuliano et al., 2003; Hou et al., 2016). This evolutionarily conserved non-heme iron enzyme family exhibits substrate promiscuity, with substrate isomerization sometimes required for oxidation (McQuinn et al., 2015). In Arabidopsis, nine CCD family members demonstrate functional specialization: NCEDs convert 9-cis-violaxanthin/neoxanthin to xanthoxin (ABA precursor) (Nambara and Marion-Poll, 2005); CCD7/8 sequentially cleave substrates for strigolactone biosynthesis (Alder et al., 2012); while CCD4 isoforms generate flavor/aroma compounds in tissue-specific patterns (Rubio-Moraga et al., 2014). Gene duplication events, exemplified by the discovery of CCD2 in Crocus sativus, drive functional diversification within the CCD family (Frusciante et al., 2014). Cleavage site preferences of various CCDs have been well defined: CCD1 targets 9.10 positions, CCD2 specifically cleaves at 7,8 sites near β-ionone rings, while CCD4 exhibits dual positional specificity (Vega-Teijido et al., 2019).

Nonenzymatic oxidation occurs primarily under oxidative stress, where reactive oxygen species drive nonspecific carotenoid degradation. In chloroplast reaction centers, spatial separation prevents β-carotene from quenching excited chlorophyll (³Chl), leading to singlet oxygen (¹O_2_) generation that randomly oxidizes carotenoid backbones into volatile aldehydes and ketones (Ramel et al., 2012a). Lipoxygenases like TomLoxC in tomato further contribute to this process by oxidizing β-carotene despite their primary role in fatty acid metabolism (Hayward et al., 2017; Gao et al., 2019).

Collectively, these pathways demonstrate that β-CC originates both as a regulated metabolic signal and as a stress-induced oxidation product. The evolutionary conservation of core CCD enzymes across plant lineages, particularly the CCD4 and NCED clades, suggests broad potential for cross-species applications, though functional divergence in non-model species necessitates careful examination. An overview of β-carotene-derived apocarotenoids, their associated cleavage enzymes, and their characterized biological roles across taxa is summarized in **Table 1**.

**Table 1:** β-Carotene-derived apocarotenoids, associated CCD enzymes, biological functions, and species where relevant genes have been characterized.

Apocarotenoid	CCD enzymes	Biological functions	Functionally characterized genes and species	References
Abscisic acid	NCEDs	A well-known phytohormone involved in coordinating various developmental and physiological process such as abiotic and biotic stress response, seed dormancy induction, stomatal closure to limit water loss, and modulation of root architecture for optimized nutrient uptake.	Maize = *vp14* Tomato = *notabilis* Arabidopsis = *Atnced3*	(Nambara and Marion-Poll, 2005; Chen et al., 2020)
Strigolactones	CCD7, CCD8	A class of carotenoid-derived phytohormones, essential in regulating root architecture development and mediation of essential biotic interactions by facilitating symbiotic relationships with arbuscular mycorrhizal fungi (AMF) and acting as germination stimulants for parasitic plants under the Orobanchaceae family.	Rice = *HTD1* (CCD7); *D10* (CCD8) Arabidopsis = MAX3 (CCD7); MAX4 (CCD8);	(Koltai, 2011; Ruyter-Spira et al., 2013; Sun et al., 2016; Wu et al., 2022)
β-Cyclocitral	CitCCD4b CsCC4	A cyclic volatile apocarotenoid that has multiple stress-signaling functions by regulating oxidative stress responses and modifying root growth prioritization. It also serve as an herbivore-induced plant volatile that primes defense mechanisms against insect herbivores. In lower photosynthetic organisms, such as cyanobacteria and mosses, it has cell-to-cell communication function.	*Citrus paradise* = *CitCCD4* Saffron = *CsCCD4*	(Frusciante et al., 2014; Zheng et al., 2021a)
β-Ionone	CCD1, CCD4, CCD7	A cyclic volatile apocarotenoid that mediates critical ecological interactions by attracting pollinators and seed-dispersing frugivores. It can also function as a plant defense metabolite, exhibiting potent repellent activity against herbivorous pests such as spider mites (*Tetranychus* spp.) and flea beetles (*Phyllotreta* spp.), deterring both feeding and oviposition.	Peach = *PpCCD4* *Dendrobium officinale* = *DoCCD1* *Forsythia suspensa* = *FsCCD4* *Cerasus humilis* = *ChCCD1* Maize = *ZmCCD7*	(Liang et al., 2021; Simkin, 2021; Felemban et al., 2024)

Because β-CC formation is tightly coupled to carotenoid turnover and reactive oxygen dynamics, its synthesis links photosynthetic metabolism directly to stress perception. This biochemical connection provides the foundation for its role as a mobile signaling molecule that conveys chloroplast-derived oxidative cues to other cellular compartments. Understanding how these biosynthetic events translate into downstream signaling is therefore essential for elucidating the physiological relevance of β-CC. The following section discusses current evidence for β-CC-mediated signaling under abiotic stress and its emerging role in coordinating plant adaptive responses.

## 3 β-CC as a signaling molecule under abiotic stress

β−CC elicits diverse stress-responsive signals, enhancing plant survival under unfavorable conditions. A central component of abiotic stress responses is the production and accumulation of reactive oxygen species (ROS). While traditionally viewed as toxic byproducts of stress, ROS are now recognized as key signaling molecules that mediate retrograde signaling, where chloroplast-originated signals influence nuclear gene expression.

In Arabidopsis, hydrogen peroxide (H_2_O_2_) production via NADPH oxidases initiates signaling cascades from the plasma membrane that ultimately trigger antioxidant defenses (Ma et al., 2012; Ben Rejeb et al., 2015a, 2015b). Calcium influx and the generation of an electrochemical gradient further amplify ROS signals throughout the plant (Gilroy et al., 2016). This leads to the activation of stress-responsive transcription factors, including members of the WRKY and MYB families (Mabuchi et al., 2018; Naing and Kim, 2021; Zheng et al., 2022; Javed and Gao, 2023). Notably, singlet oxygen (^1^O_2_), one of the most reactive forms of ROS, is unlikely to act directly as a signaling molecule due to its extremely short half-life and membrane impermeability. β−CC has been identified as a likely secondary messenger, transmitting the signal initiated by ^1^O_2_.

Recent studies support the role of β−CC as a retrograde signal that can migrate from the chloroplast to other cellular compartments (Chi et al., 2015; Chan et al., 2016; D’Alessandro and Havaux, 2019). Other retrograde signaling molecules, such as 3′-phosphoadenosine 5′-phosphate (PAPS) and dihydroxyacetone phosphate (DHAP), require specific membrane transporters (Vogel et al., 2014; Wang et al., 2020; Ashykhmina et al., 2022). In contrast, β−CC is a small, volatile, and lipid-soluble molecule that can potentially diffuse freely across membranes. This property allows β−CC to function as a mobile signal during abiotic stress responses. Nevertheless, the precise mechanism by which β−CC is transported, and the identity of its primary molecular targets remain unclear.

**Figure 2:**
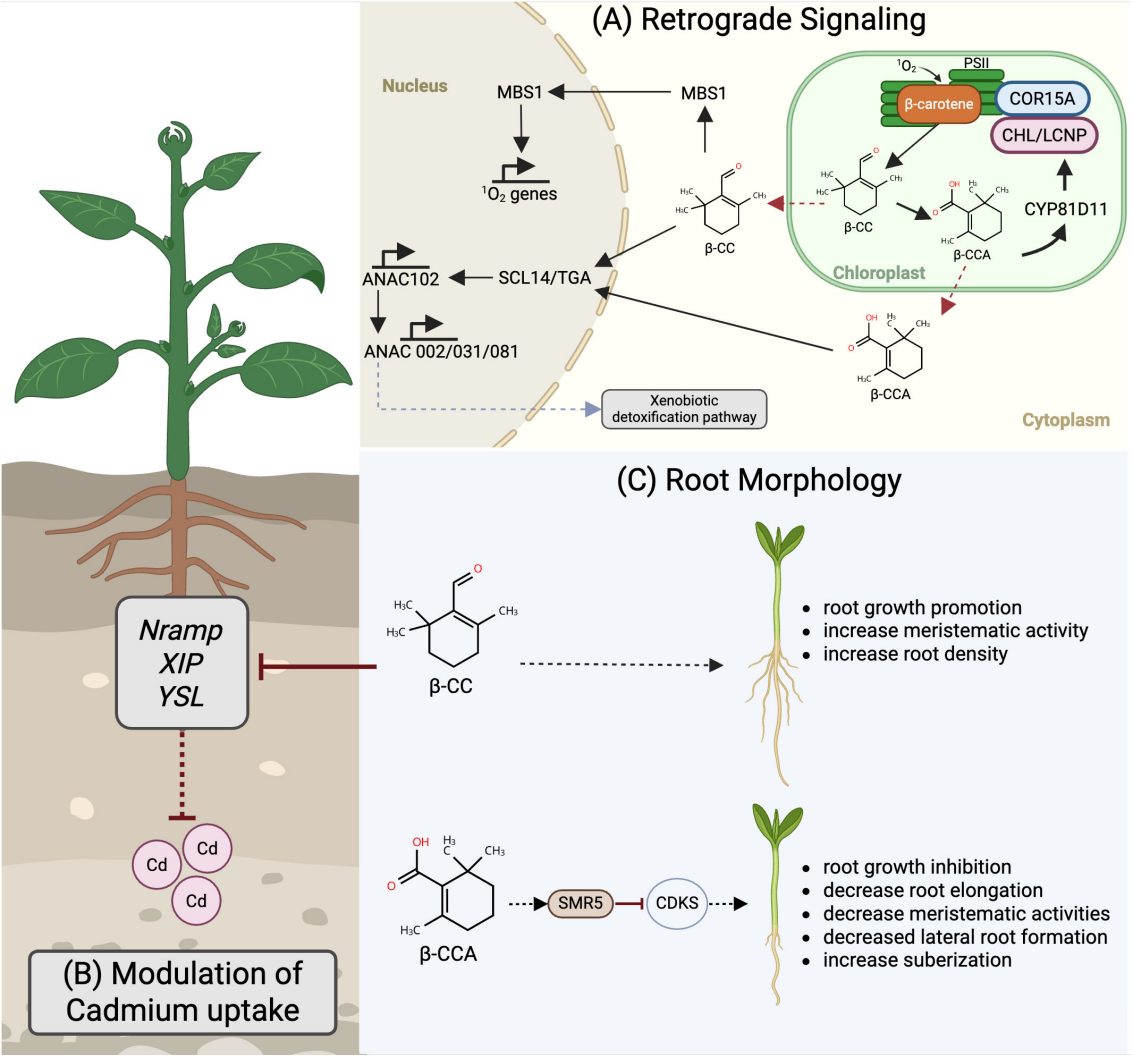
Summary of the effects of β-CC in plants under different abiotic stresses. Several studies have elucidated the role of the apocarotenoid β-CC in different abiotic stress conditions. **(A)** Retrograde signaling in photooxidative and drought stress: β-CC activates chloroplast-to-nucleus signaling by inducing MBS1 and SCL14/TGA transcription factors, triggering the xenobiotic detoxification pathway. MBS1 influences singlet oxygen (^1^O_2_-responsive genes, whereas SCL14 upregulates ANAC transcription factors, mitigating oxidative damage. Under drought stress, the water-soluble derivative β-CCA activates SCL14-dependent detoxification and modulates the cell cycle regulator SMR5, balancing stress tolerance with growth trade-offs. **(B)** Cadmium (Cd) stress mitigation: β-CC suppresses Cd uptake by downregulating the expression of symplastic transporters (Nramp, ZIP, and YSL) and enhances glutathione-mediated sequestration. **(C)** Root morphology regulation: β-CC promotes root growth via meristematic activation and increases lateral root density, whereas β-CCA inhibits elongation, reduces lateral roots, and enhances the development of a protective layer via suberization and lignification. Created in BioRender. Lachica, G. (2025) https://BioRender.com/a87becd.

**Figure 2** summarizes the current understanding of β-CC-mediated responses under different abiotic stress conditions, as well as its regulatory influence on root development. The following subsections discuss these processes in detail, emphasizing the molecular and physiological mechanisms through which β-CC contributes to plant stress tolerance.

### 3.1 Photooxidative stress

Photooxidative stress, caused by excess light energy under high irradiance or fluctuating environmental conditions, poses a serious threat to global crop productivity. When photosynthetic capacity is overwhelmed, chloroplasts produce elevated levels of ROS, particularly ^1^O_2_, leading to lipid peroxidation, chloroplast damage, and irreversible photoinhibition (Foyer, 2018). Crops such as wheat, rice, and maize are especially vulnerable to light-induced yield loss, with potential reductions of 20–30% under extreme conditions.

Plants have evolved photoprotective mechanisms, including non-photochemical quenching (NPQ) and enzymatic ROS scavenging. In addition to these, recent studies highlight the additional role of β-CC as a key retrograde signal that adjusts gene expression under light stress. β-CC accumulation has been observed in *Arabidopsis* leaves exposed to high-light conditions, resulting in extensive transcriptional reprogramming that shifts cellular priorities from growth to defense (Ramel et al., 2012b). Upregulated genes are associated with oxidative stress response, detoxification, and hormone signaling, whereas genes related to cell development and biogenesis are typically downregulated (Ramel et al., 2012b). These transcriptional profiles overlap substantially with those induced by ^1^O_2_, supporting the role of β-CC as a downstream messenger in singlet-oxygen signaling.

Two main signaling routes mediate β-CC-dependent photoprotection. The first involves the zinc-finger protein Methylene Blue Sensitivity 1 (MBS1), which functions downstream of β-CC and is essential for the activation of ^1^O_2_-responsive genes (Shumbe et al., 2017). In *Arabidopsis*, increased levels of MBS1 have been associated with improved tolerance to high-light stress. This effect has been demonstrated in *ch1* mutants, which overproduce ^1^O_2_, and in wild-type plants treated with exogenous β-CC (Shumbe et al., 2017). Overexpression of *MBS1* leads to hallmark photoprotective responses, including reduced lipid peroxidation, decreased levels of hydroxy fatty acids, and enhanced PSII photochemical efficiency (Shao et al., 2013; Shumbe et al., 2017). Transcriptomic analyses further demonstrate that many ROS-responsive genes are co-regulated in β CC-treated and *MBS1*-overexpressing lines, underscoring the central role of this pathway in light stress adaptation.

A second, MBS-independent route involves the GRAS family protein SCARECROW-LIKE 14 (SCL14), which modulates detoxification processes in coordination with TGA class II transcription factors (Ndamukong et al., 2007; Fode et al., 2008). β-CC treatment induces SCL14-regulated transcription factors including ANACs, leading to the activation of genes involved in xenobiotic metabolism and enhanced photoprotection, particularly in young leaves (D’Alessandro et al., 2018). This pathway expands the functional scope of β-CC signaling by linking oxidative stress mitigation with cellular detoxification.

A more recent study further broaden this framework. Tiwari et al. (2025) demonstrated that β-CC and its oxidized derivative β-CCA induce the expression of *CYP81D11*, a cytochrome P450 gene that plays a pivotal role in apocarotenoid-mediated stress tolerance. Overexpression of *CYP81D11* (OE: CYP81D11) was sufficient to confer high levels of tolerance comparable to those provided by exogenous β-CC application. Unlike the detoxification route mediated by *SCL14*, this mechanism enhances the photosynthetic capacity of leaves, improving electron transport efficiency and CO_2_ fixation under high-light conditions. The resulting increase in photosynthetic performance reduces ^1^O_2_ photoproduction and limits secondary oxidative damage, as evidenced by decreased lipid peroxidation and reactive carbonyl accumulation. *CYP81D11* overexpression also upregulates protective chloroplastic genes such as *COR15A* and *CHL/LCNP*, which stabilize plastidial membranes and prevent lipid oxidation. These results identify *CYP81D11* as a downstream effector of β-CC and the *TGAII-SCL14* signaling that complements the detoxification pathway by reinforcing photosynthetic efficiency and stabilization of plastidial structure (Tiwari et al., 2025).

Together, these findings position β-CC as a pivotal regulator of photooxidative stress responses. Its ability to modulate multiple signaling pathways highlights its versatility as a redox-responsive signal. Given the potential divergence of β-CC signaling components among plant species, comparative analyses in crops remain important for understanding how this apocarotenoid contributes to field-level stress resilience.

### 3.2 Salinity

Salinity stress, cause by the accumulation of excessive soluble salts in the soil, disrupts plant physiology by inducing osmotic imbalance, ion toxicity, and reduced nutrient uptake (Ullah et al., 2017). These disruptions manifest as stunted growth, chlorosis, and poor seed germination, ultimately leading to significant yield losses in major crops (Läuchli and Grattan, 2007; Isayenkov and Maathuis, 2019). The intensification of soil salinization has been further accelerated y anthropogenic factors such as improper irrigation practices and excessive fertilizer use, compounded by the effects of climate change (MaChado and Serralheiro, 2017; Hopmans et al., 2021).

Emerging evidence indicates that β−CC contributes to salinity by influencing root system architecture. Foliar application of β−CC has been reported to increase primary root length and lateral root density in *Arabidopsis*, rice, and tomato exposed to saline conditions (Dickinson et al., 2019). Treated plants exhibited improved vigor and biomass accumulation, suggesting a positive impact on root growth dynamics. This response appears to result from enhanced meristematic activity in both primary and lateral roots, independent of classical hormonal pathway such as auxin, brassinosteroid, or ROS signaling (Dickinson et al., 2019).

While this response has been consistently observed in several plant species, the generality of β-CC-induced root enhancement across different crops remains to be verified. Reported effects appear to vary depending on concentration, developmental stage, and experimental conditions, suggesting that β-CC’s impact on root growth may be context dependent. Further studies are needed to establish whether this growth response represents a conserved signaling outcome or a species-specific physiological adjustment.

Understanding the extent to which β-CC contributes to root system plasticity under salinity will be critical for determining its broader significance in plant stress adaptation and its potential as a tool for improving crop resilience.

### 3.3 Heavy metals

Heavy metals play dual roles in plant systems, where trace amounts of micronutrients such as zinc and copper are essential for metabolic function, yet excessive accumulation causes severe phytotoxicity. With rapid industrialization in recent decades, heavy metal contamination in agricultural soils has significantly increased, posing serious threats to crop production and food safety. Cadmium (Cd) is particularly problematic because of its high solubility and mobility in soil, which promote rapid uptake by roots and subsequent entry into the food chain (Wang et al., 2023; Song et al., 2025). Cd toxicity manifests through multiple physiological disruptions, including oxidative stress via ROS generation, displacement of essential metal cofactors in enzymes, and interference with mineral nutrition (Stohs and Bagchi, 1995; Pourrut et al., 2011; Shahid et al., 2014; Mansoor et al., 2023). These biochemical disturbances lead to visible symptoms such as growth retardation, leaf chlorosis, and yield loss.

Plants have evolved sophisticated defense mechanisms against Cd toxicity that function at both preventive and remedial levels. The first line of defense involves exclusion mechanisms at the root–soil interface, where specialized transport systems regulate metal uptake (Lux et al., 2011; Zhang et al., 2024). Once Cd enters the plant, intracellular detoxification pathways are activated. Enzymatic antioxidants such as peroxidase, superoxide dismutase, and ascorbate peroxidase, mitigate oxidative damage (Xu et al., 2008; Jing et al., 2021; Luo and Zhang, 2021; Liu et al., 2024; Noor et al., 2024). Concurrently, nonenzymatic systems, particularly glutathione-mediated chelation, facilitate the vacuolar sequestration of Cd ions, isolating them from sensitive cellular components (Hsu and Kao, 2007; Xiong et al., 2023).

β-CC has recently been implicated in enhancing plant tolerance to heavy-metal stress. Application of β-CC has been shown to reduce Cd accumulation in plant tissues and to alleviate toxicity symptoms, as demonstrated in quinoa (Sun et al., 2021). This protective effect involves several complementary mechanisms. β-CC enhanced antioxidant capacity, modulates the expression of Cd-transporter genes (*Nramp, ZIP, and YSL*), and promotes cellular redox balance. By strengthening detoxification systems while simultaneously and limiting Cd uptake, β-CC treatment improves root growth, biomass accumulation, and overall plant vigor under Cd stress. These findings suggest β-CC may serve as a promising molecule for improving heavy-metal tolerance and could be explored further for phytoremediation applications in contaminated environments.

### 3.4 Drought

Agricultural drought, characterized by prolonged rainfall deficits and depletion of soil water reserves, remains as one of the most significant constraints on global crop productivity. This complex stress system accounts for more than 40% of productivity losses worldwide between 2007 and 2022 (FAO, 2023). Climate projections indicate that drought frequency and intensity will continue to increase, particularly threatening the rainfed agricultural systems that account for the majority of global cultivated land (Chiang et al., 2021; Qi et al., 2022; Vieira and Stadnyk, 2023).

Plants respond to drought through a range of adaptive strategies that can be broadly classified into avoidance and tolerance mechanisms (Fang and Xiong, 2014). Avoidance strategies include morphological adjustments that maintain water uptake and reduce water loss, such as enhanced root development, altered leaf morphology, and accelerated developmental transitions (Cruz de Carvalho, 2008; Butt et al., 2017; Ke et al., 2022). Tolerance mechanisms, on the other hand, operate at the cellular and molecular levels through osmolyte accumulation, activation of antioxidant system, and transcriptional reprograming via both ABA-dependent and ABA-independent pathways.

The apocarotenoid β-CC acts as a key mediator of drought responses through non-classical signaling mechanisms (Deshpande et al., 2021b). β-CC pretreatment significantly improves drought resilience in tomato, with treated plants maintaining higher relative water content under identical stress conditions. This protective effect is associated with enhanced stomatal regulation, increased proline accumulation through *p5cs* upregulation, and elevated osmoprotective amino acid levels. Notably, these effects occur independently of the classical ABA signaling, as similar responses are observed in ABA-insensitive lines.

At the cellular level, β-CC contributes to oxidative stress management during drought. Treated plants exhibit increased superoxide dismutase activity, reduced lipid peroxidation, and sustained photosynthetic efficiency under water deficit conditions (Deshpande et al., 2021a). These biochemical adjustments are accompanied by altered carbon allocation patterns that favor root system development and enhanced photosynthate partitioning to underground tissues. This aligns with earlier findings showing β-CC-induced root stimulation under salt stress by Dickinson et al. (2019), suggesting a broader regulatory role for β-CC in coordinating growth and stress adaptation.

The oxidized derivative β-cyclocitric acid (β-CCA) mediates additional drought adaptation pathways through distinct but complementary mechanisms. Water-soluble β-CCA accumulates preferentially in leaf tissues, where it activates drought- and β-CC-responsive genes, including *ATAF1*, *RD29A*, *RD22*, *RD26*, and *bZip60* (D’Alessandro et al., 2019). In *Arabidopsis*, ATAF1, a NAC transcription factor, regulates drought adaptation through both ABA-dependent and ABA-independent pathways. It activates *NCED3* to promote ABA biosynthesis but can also repress stress-responsive genes, as *ataf1* mutants recover more effectively than wild type (Lu et al., 2007; Jensen et al., 2013). *ATAF1* expression is enhanced in ABA-deficient backgrounds, linking it to ROS-mediated signaling (Wu et al., 2009). *RD29A* reflects connections to classical stress pathways, while the NAC transcription factors *RD22* and *RD26* coordinate developmental and metabolic responses to dehydration (Shinozaki and Yamaguchi-Shinozaki, 2007; Singh et al., 2021). In addition, *bZIP60* contributes to endoplasmic reticulum and osmotic stress responses by maintaining proteostasis under water deficit (Yang et al., 2025).

The induction of these genes by both β-CC and β-CCA suggests that apocarotenoid signaling converges with canonical drought pathways while extending its influence on oxidative stress regulation and transcriptional control. Transcriptome analyses reveal substantial overlap between β-CC-responsive and ^1^O_2_-responsive gene networks, supporting a chloroplast-to-nucleus retrograde signaling route (Ramel et al., 2012b; D’Alessandro et al., 2019; Braat et al., 2023). Within this framework, β-CCA functions through the MBS1-SCL14 axis, where MBS1 transduces singlet-oxygen-related cues an SCL14 coordinates the downstream activation of xenobiotic detoxification genes, including glutathione S-transferases, UDP-glycosyltransferases, and cytochrome P450s (Shumbe et al., 2017; D’Alessandro et al., 2018).

Comparative studies indicate that both β-CC and β-CCA can initiate this MBS1–SCL14 cascade under drought. However, β-CCA extends its regulatory role to cell-cycle control through the induction of *SMR5*, a cyclin-dependent kinase inhibitor (Braat et al., 2023; Braat and Havaux, 2024). This bifurcation allows plants to simultaneously manage oxidative stress and developmental reprogramming. One pathway engages SCL14-mediated detoxification, while the other suppresses cell division via SMR5, leading to reduced lateral root initiation and enhanced suberization. These coordinated changes promote water conservation and tissue protection during prolonged drought.

The physiological relevance of these mechanisms has been validated across multiple species. In *Arabidopsis*, β-CCA markedly improved survival and post-drought recovery, with treated plants remaining turgid during water withdrawal and fully recovering after rehydration. Treated leaves maintained higher relative water content (RWC) and exhibited reduced electrolyte leakage, reflecting improved membrane integrity. The protective effect was closely linked to *SMR5* induction, which down-regulates cell-cycle activators while upregulating water deprivation and detoxification genes, leading to enhanced root suberization and reduced nonstomatal transpiration (Braat et al., 2023).

In *Prunus persica* (peach), β-CCA treatment significantly enhanced drought tolerance by alleviating leaf wilting and promoting rapid recovery after rewatering (Zhu et al., 2023). Treated seedlings maintained higher leaf RWC and lower electrolyte leakage, reflecting improved membrane stability and water retention. β-CCA increased root activity and the number of root tips, suggesting enhanced capacity for water uptake, and preserved photosynthetic efficiency through higher chlorophyll fluorescence and net photosynthetic rate. Transcriptomic data confirmed the upregulation of photosystem and Calvin cycle genes, together with antioxidant and detoxification pathways including *GST*, *SCL14*, and *ANAC102*. These physiological and molecular responses were accompanied by activation of genes related to cutin, suberine, and wax biosynthesis, as well as modulation of cytokinin and brassinosteroid metabolism. Complementary findings show that both β-CC and β-CCA induce *CYP81D11*, a cytochrome P450 gene that enhances photosynthetic capacity and mitigates ROS production under water deficit (Tiwari et al., 2025). Collectively, these results demonstrate that β-CCA functions as a potent, non-ABA signaling molecule that enhances drought resilience by sustaining photosynthesis, preserving water status, and activating antioxidant and structural defense mechanisms.

In *Solanum lycopersicum* (tomato), β-CC acts as a volatile precursor that primes plants for drought tolerance through ABA-independent mechanisms (Deshpande et al., 2021a, 2021b; Deshpande and Mitra, 2023). Drought triggers ROS-mediated oxidation of β-carotene, leading to β-CC accumulation, while exogenous β-CC treatment enhances stress resilience even before drought onset. Treated plants maintained higher relative water content and exhibited reduced ROS accumulation compared with controls, supported by increased proline concentration and elevated SOD activity. β-CC also protected the photosynthetic machinery, resulting in a twofold increase in net photosynthetic rate and a sixfold rise in total chlorophyll content under drought stress. The additional carbon gained was preferentially allocated to below-ground tissues, producing roots with 52% greater dry mass and 39% longer total length than in untreated plants (Deshpande et al., 2021a). These findings confirm β-CC as a growth-promoting and drought-priming signal that enhances redox homeostasis, photosynthetic efficiency, and root system vigor under water deficit.

Beyond these systemic and species-specific effects, β-CC and β-CCA exert opposing influences on root architecture that underpin their drought responses. β-CC functions as a conserved promoter of root growth across Arabidopsis, rice, and tomato, and (Dickinson et al., 2019; Deshpande et al., 2021a). It enhances primary-root elongation and lateral-root branching through increased meristematic cell division and up-regulation of cell-cycle markers such as CYCB1;1, independent of hormonal signaling. The resulting deeper and more compact root systems improve water and nutrient acquisition under stress. In contrast, β-CCA suppresses cell elongation and division through *SMR5* induction and the downregulation of cyclins and CDKs (Braat et al., 2023). This response limits lateral-root initiation while enhancing suberization, redirecting metabolic resources from growth toward defense and water conservation.

The strength and nature of these responses vary among species and experimental conditions. In *Arabidopsis*, β-CCA strongly suppresses root elongation even in the absence of water stress, consistent with its role as a stress-induced growth inhibitor (Braat et al., 2023). In *Prunus persica* seedlings, however, β-CCA treatment does not significantly affect total root length or surface area but enhances root activity and increases the number of root tips under drought (Zhu et al., 2023). These differences likely reflect both species-specific growth dynamics and environmental factors influencing β-CC oxidation and β-CCA accumulation. Braat et al. (2023) demonstrated that under certain conditions, volatile β-CC can be rapidly converted in planta into β-CCA, leading to root inhibition rather than stimulation. This conversion appears sensitive to growth media composition and sucrose levels, suggesting that environmental modulation of β-CC uptake and oxidation may determine the direction of the root response. Conversely, in slower-growing woody species such as peach, β-CCA accumulation primarily enhances physiological root activity rather than imposing morphological inhibition (Zhu et al., 2023).

Collectively, the evidence establishes β-CC and β-CCA as central components of an integrated apocarotenoid signaling network that coordinates stress perception, detoxification, and developmental plasticity during drought. Through the MBS1/SCL14 pathway, both compounds relay oxidative cues from the chloroplast to the nucleus, activating xenobiotic detoxification and antioxidant defenses. In parallel, induction of CYP81D11 strengthens photosynthetic capacity and limits ROS generation, while SMR5-mediated control of the cell cycle redirects developmental resources toward tissue protection and water conservation. The opposing actions of β-CC-driven root promotion and β-CCA-induced growth restraint exemplify a finely tuned balance between resource acquisition and defense maintenance. Together, these coordinated molecular and physiological processes define a conserved drought-response module in which β-CC primes growth and redox stability, and β-CCA consolidates tolerance through cellular reprogramming and structural fortification.

## 4 Translational perspectives: leveraging β-cyclocitral signaling insights for crop improvement

The signaling functions of β-CC and its oxidized derivative β-CCA have been characterized across diverse plant taxa, including *Arabidopsis*, rice, tomato, and peach. These studies collectively reveal conserved pathways that link carotenoid oxidation with stress signaling, redox regulation, and developmental reprogramming. The apparent conservation of β-CC–mediated pathways across monocots and dicots suggests that similar mechanisms may operate in other crops where this signaling has yet to be explored. Among these, soybean (*Glycine max*) represents a particularly promising system for translational application. As a globally important legume highly sensitive to drought, salinity, and photooxidative stress, soybean provides an ideal model for testing whether apocarotenoid signaling can be harnessed to enhance abiotic stress tolerance in economically relevant species.

Soybean (*Glycine max* L. Merr.) ranks among the world’s most widely cultivated legumes, producing over 370 million metric tons annually across 136 million hectares (FAO, 2025). Beyond its role as a major source of plant protein and oil, soybean contributes to agricultural sustainability through symbiotic nitrogen fixation, improving soil fertility and reducing dependence on synthetic fertilizers (Liu et al., 2021; Alves et al., 2024; Yan et al., 2024). However, soybean growth and productivity are highly sensitive to environmental fluctuations. Photoperiod, temperature, and water availability remain key determinants of yield potential (Major and Johnson, 1974; Major et al., 1975b, 1975a). Moreover, a large proportion of global soybean cultivation is rainfed, making the crop especially vulnerable to climate variability (Barrera et al., 2025). Drought stress during reproductive stages can result in yield losses exceeding 70–80% when water deficits coincide with flowering and pod filling (Poudel et al., 2021; Nguyen et al., 2023; Ferreira et al., 2024). With increasing drought frequency and intensity projected under future climate scenarios, developing soybean cultivars with improved stress resilience is an urgent priority.

Traditional breeding has enhanced yield and quality traits, but the polygenic and environment-dependent nature of abiotic stress tolerance has limited rapid genetic gains. Complex genotype-by-environment interactions, coupled with the slow progress of conventional selection, have prompted growing interest in biotechnological and physiology-based approaches that leverage new insights into plant signaling and metabolism (Mohapatra et al., 2024; Mushtaq et al., 2025; Prasanna et al., 2025; Vieira et al., 2025). In this context, β-CC has emerged as a potential signaling molecule of agronomic relevance due to its ability to modulate key stress response networks in model species.

Mechanistic studies have shown that β-CC enhances drought tolerance independently of classical ABA signaling while modulating root system architecture through pathways partially analogous to those of SLs. Its oxidized derivative, β-CCA, adds another regulatory layer through activation of the SCL14-dependent detoxification pathway and modulation of cell-cycle regulators such as the cyclin-dependent kinase inhibitor SMR5 and downstream ANAC transcription factors (D’Alessandro et al., 2019; Braat et al., 2023). Comparative genomic analyses indicate that soybean possesses orthologs corresponding to many of these regulatory genes identified in Arabidopsis and other species (**Table 2**). The functional characterization of these orthologs, such as *GmMBS1*, *GmSCL14*, *GmSMR5*, and *GmCYP81D11*, remains an important gap and represents a critical next step toward translating β-CC and β-CCA signaling research into legume systems.

**Table 2:** Soybean orthologs of Arabidopsis genes associated with β-cyclocitral and β-cyclocitric acid-mediated abiotic stress responses.

Gene	Arabidopsis Gene ID	Abiotic stress	Soybean Match				
			Phytozome gene identifier	NCBI accession	Description	E value	Percent identity
MBS1	At3G02790	photooxidative stress	Glyma.09G250600	XM_003534465.4	PREDICTED: Glycine max protein METHYLENE BLUE SENSITIVITY 1 (LOC100784149)	5.00E-25	73.13%
SCL14	AT1G07530	photooxidative stress and drought	Glyma.12G062200	XM_006592132.4	PREDICTED: Glycine max uncharacterized protein (LOC100815586)	2.00E-113	70.34%
			Glyma.11G138600	XM_003539035.5	PREDICTED: Glycine max SCARECROW-like protein 14 (LOC100796903)	3.00E-111	69.71%
ANAC102	AT5G63790	photooxidative stress and drought	Glyma.04G208300	NM_001354136.1	Glycine max NAC transcription factor (LOC100814504)	7.00E-106	77.40%
			Glyma.06G157400	NM_001255948.2	Glycine max NAC transcription factor (NAC018)	3.00E-98	76.10%
CYP81D11	AT3G2874	photooxidative stress and drought	Glyma.16G149300	XM_003547995.5	PREDICTED: Glycine max cytochrome P450 81E8 (LOC100811727)	1.00E-81	71.88%
			Glyma.11G051800	XM_006590580.4	PREDICTED: Glycine max cytochrome P450 81E8 (LOC100804509)	2.00E-57	67.29%

Despite the central role of carotenoid metabolism in plant stress physiology, carotenoid cleavage dioxygenases (CCDs) remain underexplored in soybean. Current evidence primarily concerns GmCCD1 and GmCCD4, whose expression has been linked to lutein turnover, floral pigmentation, and responses to abiotic stresses including salinity and heat (Kanamaru et al., 2009; Wang et al., 2013; Gao et al., 2020). GmCCD4 shares structural features with CsCCD4 from *Crocus sativus*, but its possible involvement in generating β-CC or related apocarotenoids under stress remains unknown. Systematic functional and biochemical analyses of the soybean CCD family will therefore be essential for elucidating the enzymatic sources of β-CC and clarifying whether its biosynthesis mirrors that observed in other crops.

Beyond genetic and molecular avenues, β-CC and β-CCA also hold potential as exogenous treatments or seed-priming agents to improve early seedling vigor under stress. Studies in model plants demonstrate that β-CC pretreatment enhances germination and seedling establishment under osmotic or oxidative stress, indicating possible utility for improving soybean emergence and early growth in drought-prone environments (Dickinson et al., 2019; D’Alessandro et al., 2019; Deshpande et al., 2021a; Braat et al., 2023). Recent work by Hazra et al. (2025) further strengthens this concept by showing that β-CC acts as a signaling molecule that extends seed longevity in *Arabidopsis*. Their results revealed that apocarotenoids, rather than carotenoids themselves, govern seed viability: double mutants lacking CCD1 and CCD4 displayed sharply reduced longevity, whereas exogenous β-CC application restored viability even under accelerated aging. β-CC treatment reduced lipid peroxidation and hydrogen-peroxide accumulation while enhancing antioxidant enzyme activity, including ascorbate peroxidase and catalase. Notably, β-CC and β-ionone directly activated expression of the tonoplast aquaporin TIP2;2, a protein essential for ROS detoxification during seed storage. Together, these findings demonstrate that β-CC contributes to seed vigor and redox stability through apocarotenoid-dependent signaling rather than pigment accumulation, highlighting its potential as a natural seed-conditioning compound for enhancing germination and longevity under adverse conditions.

Furthermore, β-CC’s influence on root architecture is of particular relevance to leguminous crops. Root growth and nodule formation are central to symbiotic nitrogen fixation, yet both processes are highly sensitive to drought and salinity (Singleton and Bohlool, 1984; Duzan et al., 2004; Kunert and Vorster, 2020; Lumactud et al., 2023). β-CC’s reported capacity to promote root branching and elongation under stress suggests potential to maintain nodulation and nitrogenase activity when water availability is limited. Future research should evaluate how β-CC treatments affect root exudation profiles, nodule initiation, and nitrogen assimilation efficiency under abiotic stress, integrating these physiological responses with molecular markers of apocarotenoid signaling.

While the economic feasibility of β-CC and β-CCA application in field conditions remains to be determined, their biological activity at nanomolar to micromolar concentrations and natural occurrence suggest a low risk of harmful residues. Nonetheless, the lack of residue data in edible tissues represents a critical knowledge gap. Compared with synthetic hormone analogs such as ABA derivatives, β-CC offers a potentially safer and more sustainable approach. Advances in microbial production systems, such as β-CC biosynthesis in engineered cyanobacteria, could further reduce costs and environmental impact, though technical scale-up and regulatory validation remain key challenges before commercial deployment.

## 5 Challenges and future directions

Despite significant advances in our understanding of carotenoid-mediated stress responses, substantial gaps remain that hinder the translation of apocarotenoid biology, particularly β−CC and its derivative β−CCA, into agricultural applications. One of the foremost challenges is the incomplete elucidation of β-CC signaling mechanisms. While its association with detoxification pathways and developmental reprogramming under abiotic stress is increasingly evident, the identity of its receptors, the specificity of its tissue-level responses, and the extent of its interaction with canonical phytohormones such as ABA, SLs, brassinosteroids, salicylic acid, and ethylene remain unresolved. A multiomics framework that integrates transcriptomic, proteomic, and metabolomic analyses, coupled with CRISPR-based functional validation, will be essential to clarify these hierarchical relationships and decode the broader β-CC–responsive network across plant systems.

The lack of translational validation of β−CC in economically important crops is also critical. To date, functional characterizations of β−CC and β−CCA have been conducted in *Arabidopsis* a few model or horticultural species, with limited extension to cereals, legumes, and other agronomically important plants. Although homologs of β−CC-responsive genes are widely conserved, including those encoding MBS1, SCL14, SMR5, and CYP81D1, their specific roles in crop stress adaptation remain to be tested under field conditions. Similarly, the CCD and SMR gene families which are central to β−CC biosynthesis and signaling have been only partially explored in crops such as soybean, maize, and wheat, leaving key regulatory nodes functionally uncharacterized.

Developing scalable and field-applicable delivery systems for β CC remains a significant challenge. Current methods of exogenous application are largely experimental and constrained by the compound’s volatility, short half-life, and instability under environmental conditions. To fully harness β CC’s potential, especially its pronounced effects on root architecture, more targeted and sustainable delivery strategies must be developed. Rhizosphere-focused approaches are particularly promising. For instance, algal biofilms have been explored as biodegradable soil amendments capable of delivering bioactive compounds directly to the root zone while simultaneously improving soil structure and microbial diversity (Gonçalves et al., 2023; Gurau et al., 2025). Likewise, nanoencapsulation technologies can stabilize the controlled release of β CC, while engineered microbial consortia could be designed to produce or release β CC in a root-proximal, stress-responsive manner (Moradipour et al., 2019; Balla et al., 2022; Szopa et al., 2022).

The potential impact of β-CC on legume symbioses warrants particular attention. Its documented effects on root growth and stress signaling suggest possible roles in regulating nodule initiation, function, and communication with *Bradyrhizobium* species. Given the importance of symbiotic nitrogen fixation to legume productivity and soil health, elucidating how β-CC modulates these interactions could significantly advance its application in sustainable crop management.

While β-CC and β-CCA represent versatile signaling molecules capable of enhancing plant resilience to multiple stresses, their broad physiological effects necessitate careful evaluation of developmental and ecological trade-offs. For example, modulation of root morphology may alter rhizosphere microbial community structure, influencing beneficial associations such as nitrogen fixation or mycorrhizal colonization. Moreover, genetic manipulation of CCD activity to increase β-CC production must be balanced against potential depletion of carotenoid pools essential for photoprotection and photosynthetic stability.

Ensuring that β-CC-mediated adaptations are both effective and ecologically sustainable will require multi-environment validation and long-term systems-level assessment. Field trials integrating physiological, molecular, and agronomic data should evaluate trait stability, metabolite persistence, and effects on nutrient cycling and soil microbial dynamics. Ultimately, realizing the translational potential of β-CC and β-CCA will depend on close collaboration among plant physiologists, molecular biologists, breeders, and agronomists. Through such interdisciplinary efforts, apocarotenoid signaling could become a foundation for developing climate-resilient and resource-efficient crops.

## 6 Conclusion

β-CC and its oxidized derivative β-CCA have emerged as key apocarotenoid signals that integrate oxidative stress perception, detoxification, and developmental regulation across diverse plant taxa. Their conserved roles in modulating photosynthetic stability, antioxidant defense, and root plasticity highlight their importance as central mediators of abiotic stress adaptation. The growing body of evidence underscores their potential as natural biostimulants and molecular targets for engineering stress-resilient crops. Yet, realizing this potential will require translational validation in field environments, functional characterization of key signaling components in major crops, and ecological assessments to ensure sustainability. Harnessing β-CC-mediated signaling thus represents a promising step toward developing climate-resilient, resource-efficient agricultural systems.
